# Integrating images from a moveable tracked display of three-dimensional data

**DOI:** 10.1186/s41235-017-0069-0

**Published:** 2017-08-23

**Authors:** Gaurav Shukla, Roberta L. Klatzky, Bing Wu, Bo Wang, John Galeotti, Brian Chapmann, George Stetten

**Affiliations:** 1 Radiation Oncology, University of Pennsylvania, Philadelphia, PA 19107 USA; 2 Department of Psychology, Carnegie Mellon University, Pittsburgh, PA 15213 USA; 3 Arizona State University-Polytechnic Campus, Mesa, AZ 85212 USA; 4 Eye and Ear Institute, University of Pittsburgh Medical Center, Pittsburgh, PA 15213 USA; 5 Robotics Institute, Carnegie Mellon University, Pittsburgh, PA 15213 USA; 6 Imaging & Neurosciences Center, University of Utah School of Medicine, Salt Lake City, UT 84108 USA; 7 Department of Bioengineering, University of Pittsburgh, Pittsburgh, PA 15213 USA

**Keywords:** Medical imaging, Visualization, Radiology

## Abstract

This paper describes a novel method for displaying data obtained by three-dimensional medical imaging, by which the position and orientation of a freely movable screen are optically tracked and used in real time to select the current slice from the data set for presentation. With this method, which we call a “freely moving *in-situ* medical image”, the screen and imaged data are registered to a common coordinate system in space external to the user, at adjustable scale, and are available for free exploration. The three-dimensional image data occupy empty space, as if an invisible patient is being sliced by the moving screen. A behavioral study using real computed tomography lung vessel data established the superiority of the *in situ* display over a control condition with the same free exploration, but displaying data on a fixed screen (ex situ), with respect to accuracy in the task of tracing along a vessel and reporting spatial relations between vessel structures. A “freely moving *in-situ* medical image” display appears from these measures to promote spatial navigation and understanding of medical data.

**Electronic supplementary material:**

The online version of this article (doi:10.1186/s41235-017-0069-0) contains supplementary material, which is available to authorized users.

## Significance

Contemporary medical technology makes it possible to acquire dense data from imaging of the human body. While the imaging data represent three-dimensional (3D) anatomy, physicians typically view the results as projected onto a screen of two dimensions. They are called upon to put the images together to build up a depiction of the patient’s anatomy, which is a complex cognitive process. This paper describes a novel method for displaying data obtained by 3D medical imaging, in which the user explores the data by moving a screen through space; each location in physical space reveals the corresponding medical image as if from an invisible body. An experiment demonstrates that this method can be useful in navigating through 3D medical image data and visualizing it to determine spatial relations not evident from individual slices.

## Introduction

Three-dimensional medical imaging from a number of modalities, including magnetic resonance (MR), computed tomography (CT), and positron emission tomography (PET) is in common use in contemporary clinical practice. Because 3D images are generally viewed one slice at a time on a two-dimensional (2D) display, navigation through the data set is necessary to make use of the volumetric data. Typically, this involves using a mouse or keyboard to translate through a stack of parallel slices, sequentially displaying each in turn on a 2D screen for detailed examination. While this method of display is sufficient for many applications, it requires the user to cognitively integrate a sequence of 2D images into a single 3D volume to establish anatomically relevant relationships. When moving the slice through the third dimension, and especially when changing slice orientation, the relative poses of subsequent slices can easily become confusing in terms of the overall 3D geometry of the underlying anatomy, reducing the effectiveness and accuracy of diagnosis. Evidence for the difficulty of the mental skills required can be found in the correlation between spatial abilities tests and a variety of medical specializations including surgery (Hegarty, Keehner, & Cohen, 2007) and the efforts to promote visualization ability through medical training, including computer image displays (John, 2007; Provo, Lamar, & Newby, 2002). Integration of contour segments into continuous curves can be attention demanding even when the components are simultaneously present within a 2D display (Houtkamp, Spekreijse, & Roelfsema, 2003; Jolicoeur, Ullman, & MacKay, 1986, 1991).

In response to the cognitive demands, various technologies have been developed to render 3D data onto a stationary 2D display (for example, Levoy, 1988; Lorensen & Cline, 1987). The two problems with this are that the data must first be analyzed to extract the surfaces of the structures to be displayed in two dimensions, potentially producing rendering error, and the occlusion of interior structures by the projection to a plane. Another approach is to navigate through 3D data by tracked tools (Hinckley, Pausch, Goble, & Kassell, 1994; Ware & Osborne, 1990) or 3D mouse control (Fröhlich & Plate, 2000). However, the range of motion of such devices is either small or restricted to variations in orientation, regardless of the scale of the source data. The scale problem may be alleviated by augmented reality systems using head-mounted displays and 3D perspective rendering (Kalkofen, Mendez, & Schmalstieg, 2007; Looser, Billinghurst, & Cockburn, 2004); however, the user must calibrate self-motion within the simulated space in order to form a spatially coherent representation of the data.

The approaches just reviewed reveal a common, and critical, problem with visualizing 3D medical image data, namely, the need to match two spatial coordinate systems: one provided by the device displaying the data; the other defined by the spatial coordinates of the imaged structures in the physical world. Our previous research has shown that projecting image data into the actual anatomical source location, called *in situ* presentation, improves the understanding of medical images and performance in image-guided tasks for both novices and experienced clinicians. Relative to remote fixed screens, which we term ex situ, an *in situ* display produces greater accuracy of perceived target depth and perceptually directed action (Wu, Klatzky, Shelton, & Stetten, 2005) and more generalizable learning (Wu, Klatzky, Shelton, & Stetten, 2008), as well as facilitating the integration of a complex 3D shape from 2D slices (Wu, Klatzky, & Stetten, 2010).

We propose that these effects arise because *in situ* imaging provides a common frame of reference for the on-screen images of medical data and the intrinsic structures they represent. This hypothesis is tested here with a novel approach that we call a “freely moving *in*-*situ* medical image” (FRISM). Initially, an unimpeded region of 3D space is registered to the coordinate system of the underlying image data set. A mobile screen can then be freely moved within that space, at any point of which it shows a 2D data plane corresponding to the target anatomy at the same location and orientation. The experiment below tests whether manipulating a display through what amounts to an “invisible patient” will promote the perception and spatial understanding of 3D anatomical relationships beyond what can be achieved with conventional stationary displays.

## FRISM display

The FRISM display developed as an extension of an *in situ* device for displaying tomographic data that was held in the hand and subject only to the range of hand movement, called the Sonic Flashlight. The latter consisted of a small screen mounted within the handle of a conventional ultrasound transducer that projected to a half-silvered mirror mounted above the shaft (Stetten & Chib, 2001). Looking into the mirror, the operator saw a virtual image, caused by the reflection of the ultrasound data in the mirror, and also saw through the mirror to the tip of the transducer in contact with the target surface. The combination of the virtual image and the sight of the physical surface produced a perceptual representation of the ultrasound slice floating beneath the surface, at the precise location where the sonic data were currently being obtained. This device was realized both as a clinical prototype displaying ultrasound (Chang, Amesur, Klatzky, Zajko, & Stetten, 2006) and as a tracked laboratory instrument displaying computer-generated targets (Shelton, Wu, Klatzky, & Stetten, 2007).

**Fig. 1:**
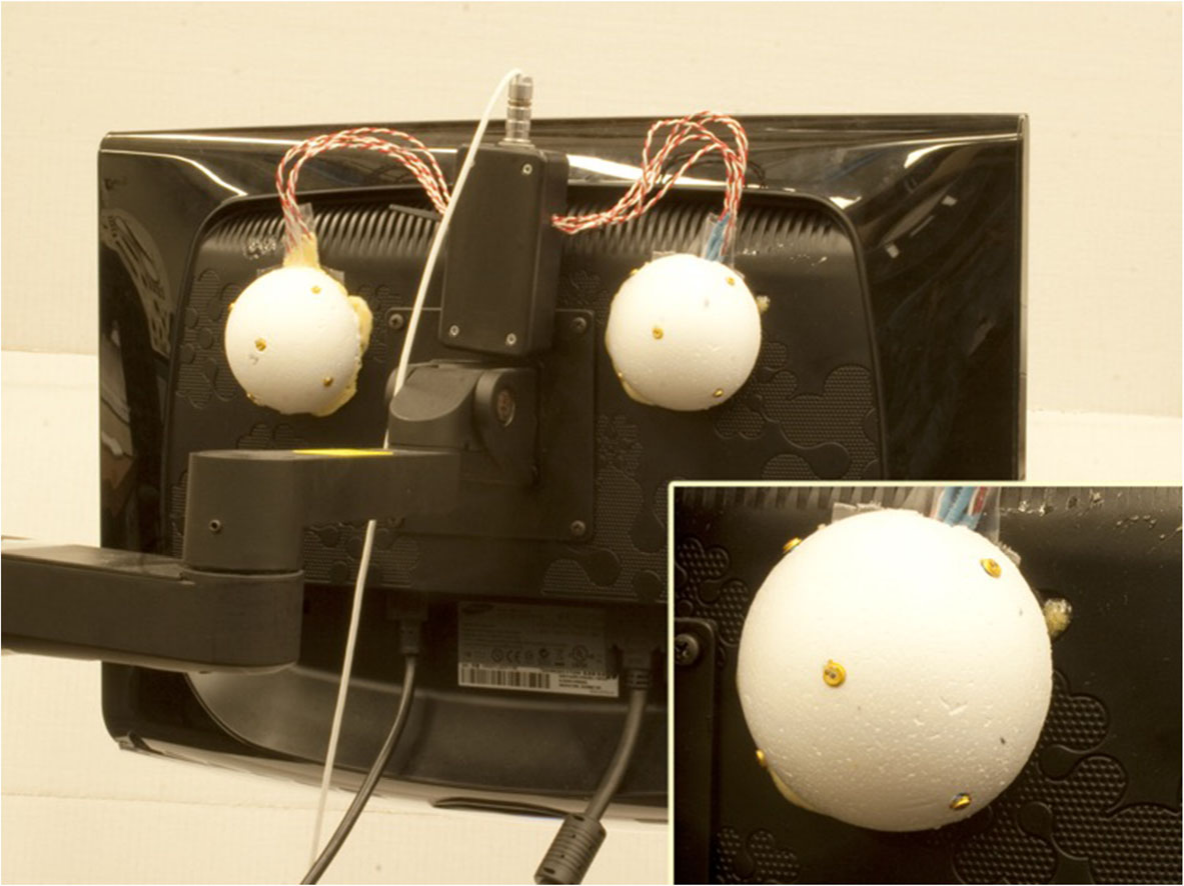
The FRISM display mounted onto its boom arm. *Inset*: close-up of infrared emitting diode marker hemisphere

**Fig. 2:**
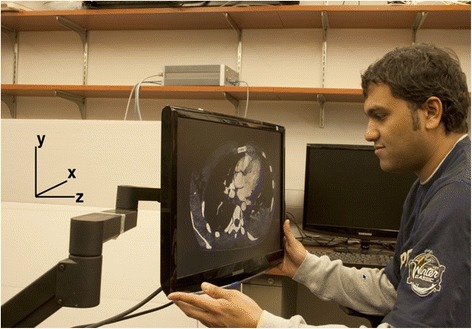
User manipulating the FRISM display, which is freely possible in six degrees of freedom including translations and rotations. A sample slice through a thoracic computed tomography scan is displayed

FRISM evolved from the hand-held apparatus, with the goal of broadening and generalizing its use by removing the half-silvered mirror and viewing the image itself on a larger screen, capable of displaying high-resolution tomographic data like that obtained from clinical CT and MR scans. The device used consisted of a 22-inch (55.88 cm) color monitor (Samsung SyncMaster 2233RZ) mounted on a moveable boom arm (see Fig. 1) that allowed the display to be manually manipulated in space in all six degrees of freedom: three translations and three rotations (see Fig. 2). Real-time tracking was accomplished using an array of ten infrared emitting diode (IRED) markers, equally divided between two 3-inch (7.62 cm) Styrofoam hemispheres rigidly mounted to the display (Fig. 1 inset), with a camera-based optical tracking system (Optotrak Certus, Northern Digital Inc.) localizing each IRED marker with an accuracy of approximately 0.1 mm and a sampling frequency of at least 100 Hz. The Optotrak software then computed orientation and position for the display as a whole, within the volume of space through which it was manipulated.

A tracked needle tool was used to calibrate the location of the four corners of the display relative to the IRED markers. A calculation was made repeatedly as the monitor was moved to render the appropriate slice from the 3D data by means of a 3D texture mapping graphical processing unit (Quadro FX 3800, NVIDIA, Inc.). The method of 3D texture mapping interpolates voxels from a 3D data set onto polygons in arbitrary planes for 2D display, in this case, a single rectangle occupying the surface of the display (Ware & Osborne, 1990). Such hardware-accelerated interpolation and projection capability is standard on high-end commercial computer graphics cards. As the 3D texture mapping system takes floating point numbers as input to define the vertices of the polygons on which the raw data voxels are interpolated, the rendering itself is inherently more accurate than the location of the IRED markers.

The CT data were anisotropic (interslice distance was more than twice the interpixel distance), but this was easily accommodated by the texture mapping hardware without additional interpolation. The 3D texture memory was simply loaded with the raw uninterpolated data, and the voxel anisotropy was addressed by scaling the physical dimension of each axis (*x*, *y*, *z*) independently during the rendering process. No extra step was required in the processing.

Coordinate transformations were defined for the virtual patient to lie within the region traversed by the movable display, such that the voxel information from a high-resolution 3D data set could be displayed as slices with arbitrary orientations and locations, as determined in real time by the physical screen location. Rendering proceeded for each video frame of the display, whether or not the user had moved the screen. (Additional file 1 shows the FRISM under test.)

## Evaluation experiment

Performance with the *in situ* device was compared to a conventional stationary display in two tasks. Novice users explored a data set derived from contrast-enhanced thoracic CT images of pulmonary vessels. In an initial navigation task, they were asked to trace along 3D vessels using 2D cross-sections and to determine where the vessels terminated. In a second task, participants reported the angular relation between two locations within the lung vasculature in the 3D space defined by the data set.

### Methods

#### Participants

Thirteen naïve observers and three coauthors (four females and twelve males), inexperienced in interpreting medical images, participated, with informed consent. All were young adults with normal or corrected-to-normal vision in both eyes.

#### Stimuli

**Fig. 3:**
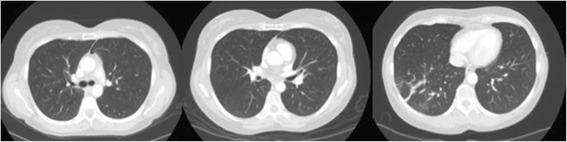
Computed tomography scans at levels of the mid-carina (branch point of the bronchi), mid-pulmonary artery, and lung base

A set of 18 contrast-enhanced CT scans of the thorax was acquired. An expert identified the pulmonary vasculature in each scan and used colored spheres (radius 5 mm) to label three structures: the pulmonary artery (PA) as it exits the right ventricle of the heart (green sphere), the left atrium (LA) where the pulmonary veins drain (blue sphere), and one distal branch of a pulmonary vessel (red sphere), which the expert recorded as objectively an artery or a vein. The spheres were visualized as cross-sectional disks overlaid on the particular slice from the data being displayed. Using each scanned CT sequence twice, once with an artery and once with a vein, a stimulus set of 36 uniquely labeled vessels was generated; each was connected to either the PA or the LA, but not both. In general, these vessels can be identified in CT images only by their anatomical connection to the heart, rather than pixel intensity or local vessel morphology, so to a novice they are indistinguishable except by tracing along the vessel, slice by slice, to either the PA or LA. Figure 3 shows representative CT images. Each scan slices across the underlying branching structure of vessels; successive slices within the volume shift which vessels are visible and the size and location of those seen continuously.

The distance (in the axial dimension anatomically of the scan) between the red sphere (marking the unknown vessel) and the correct endpoint (either the PA or LA) was used to classify each vessel into one of three categories according to its distance from the endpoint: short (5 to 30 mm), medium (50 to 80 mm), or long (>100 mm). Across stimuli, the distances were uniformly distributed among these categories and the scan was displayed at scale.

#### Design and procedure

A 3 (Distance) × 2 (Display condition: *in situ* or ex situ) within-subjects design was implemented. Here, *in situ* visualization refers to visualization of the image data on the movable FRISM display, while ex situ refers to visualization on a fixed conventional display. The ex situ display was identical to the *in situ* display, except that it was stationary on a table directly adjacent to the space in which the FRISM display would be manipulated. In either case, movement through the data was controlled by physically moving the FRISM display on its boom arm (see Fig. 2). Six trials were performed in each condition; a unique stimulus (as defined by source data and PA versus LA target) was used for each of the resulting 36 trials. Trials were blocked into two sets of 18 trials by the viewing condition, with the presentation order of trials counterbalanced across blocks and participants using a Latin square. The testing order of the two viewing conditions was also counterbalanced to avoid bias from learning.

Participants performed the experiment in a room with overhead lighting eliminated. With the screen blanked out, participants moved the FRISM display until they found the red sphere in 3D space, which appeared in cross-section as a disk on the screen when the display encountered it. The corresponding slice of CT data then appeared on the screen, with the red sphere depicted inside an unknown pulmonary vessel. Participants were instructed to remember the location of the red sphere relative to the surrounding 3D space. The first task required navigation through the data set. Participants followed the vessel by moving the display while maintaining a continuous path from the starting point to the endpoint. Eventually, the vessel terminated at a slice in which the PA (marked with a green sphere) and LA (marked with a blue sphere) both appeared, at which point the participant made a forced-choice selection about which endpoint was connected to the starting point by means of a color-coded keypad. Participants were timed during the tracing from the red sphere at the origin to the endpoint sphere (blue or green).

The second task, spatial relations, was assessed immediately after the endpoint sphere was selected. The screen was again blanked out, and participants were asked to indicate the vertical (that is, gravitationally aligned) plane containing the centers of the red starting-point sphere and the selected endpoint sphere. They responded by rotating the blank FRISM display about the *y*-axis (see Fig. 2) until it was perceived to be parallel to the vertical plane connecting the starting and endpoint locations. Valid angles ranged from −80° to +80° relative to the starting position. Prior to the experimental trials, participants performed two to four sample trials in each viewing condition to demonstrate the tasks.

### Results

#### Navigation task

The difficulty of navigation through pulmonary vasculature is limited by its intrinsic branching, which here produced a ceiling effect in performance: There were few errors in navigation (accuracy 95.8% *in situ* versus 93.1% ex situ). Although this small difference reaches the standard *p* < 0.05 significance level by 1-tail test (*t*(15) = 2.07, *p* = .028), under the directional prediction that the *in situ* display would be superior, the effect is not strong. There was also no significant difference in time to navigate (34.9 seconds for *in situ*, 38.5 seconds for ex situ). To provide a stronger test of whether the *in situ* display facilitates navigation, it would be necessary to bring performance below ceiling, possibly by constructing an artificial CT data set with more complex branching.

#### Spatial relations task

Correct and response angles were recorded as values within ± 180°, signed relative to the *z*-axis. In previous research assessing visualization of 3D relationships from 2D images, we found a type of error in which angular judgments are correct in magnitude, but reversed in direction, which was particularly prevalent with an ex situ display (Wu et al., 2010). Such “reversal errors” were clearly evident in the present data. We identified a reversal as occurring when the response differed in sign from the correct value and the absolute difference was greater than 90°. Although reversal errors were few, they followed the previous pattern that the in situ display produces fewer errors (12 versus 23 for ex situ, constituting 4.2% and 8.0% of responses, respectively); the difference reached significance by 1-tailed test, *p* < .05. To eliminate the effects of these errors in the subsequent analyses, the sign of the response angle for such trials was reversed.

The principal measure of performance on the visualization test is the degree to which the mean reported angle for a given stimulus matches the correct value for that stimulus. When response angles are regressed against actual values, the ideal slope would be 1.0. When such regressions were done for each display, the slope for FRISM was closer to the ideal than the slope for the conventional, ex situ display (mean slope = .94 for *in situ* versus .85 for ex situ, *r* ^2^ = .96 and .98, respectively, *t*(15) = 3.26, *p* = .005). (If the same test is done with the naïve participants alone, the results are essentially the same: mean = .92 for *in situ* versus .83 for ex situ, *t*(13) = 3.78, *p* = .003.)

**Fig. 4:**
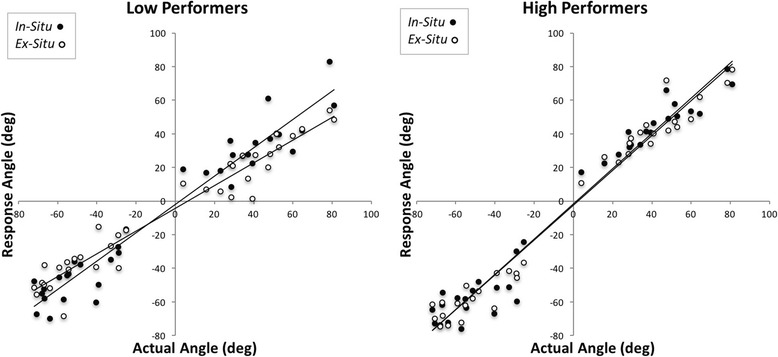
Mean response against correct angle, for participants with *below-median* and *above-median* slope values (*low* and *high performers*), by display. Least squares regressions have been fit to the data

This result is augmented by an analysis of individual performance in the spatial relations task, which divided the participants into two groups that corresponded to levels above and below the median of the slope averaged over the two displays. The relation between the response and actual angles is shown for each group and display in Fig. 4. The higher-performing group showed little advantage from *in situ* imaging, as their slopes statistically reached the ideal value of 1.0 in both cases (mean slope = 1.05 and 1.03 for *in situ* and ex situ, *r* ^2^ = .96 and .97, respectively). In contrast, the lower performers were substantially aided by the *in situ* display (mean slope = 0.84 and 0.68 for *in situ* and ex situ, *r* ^2^ = .94 and .94, respectively).

**Fig. 5:**
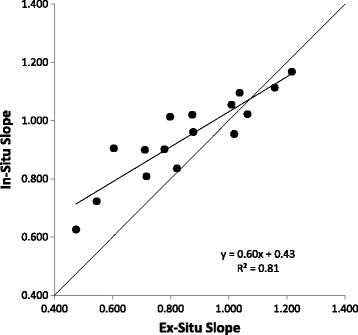
Relation between slopes of functions relating response to correct angle for individual participants in the visualization task, along with best-fit line and goodness-of-fit. The *diagonal* indicates no enhancement from *in situ* visualization

Performance with the two displays was highly correlated across individual participants, as shown in Fig. 5. Notably, the extent to which a participant’s slope for *in situ* exceeded that for ex situ (with no improvement indicated by points on the diagonal) tended to be greater, as a participant’s overall performance was poorer. In other words, *in situ* imaging provided the greatest help to those who needed it most.

Absolute errors in the reported angle between start and endpoints, which combines systematic and variable error, were analyzed with an ANOVA on modality × distance to the endpoint. Mean absolute error was reduced by *in situ* viewing (11.7° versus 14.7° for ex situ), *F*(1,15) = 6.65, *p* = .021. There was no systematic effect of distance, *F*(2,30) = 1.06 (mean = 14.36°, 12.30°, and 12.85° for short, medium, and long, respectively), nor was there a significant interaction, *F*(2,30) <1.

Note that the foregoing analyses ignore the few errors that arose in the navigation task, which could indicate a misconception of the correct angle. An alternate analysis on the spatial relations data, assigning the “correct” response angle to whichever ending location had been indicated in the navigation task, produced essentially equivalent results.

## Discussion

These results support the hypothesis that the *in situ* display of medical image data confers a benefit to naïve participants in navigating through 3D medical image data and visualizing it to determine 3D spatial relations not evident from individual slices. The *in situ* advantage was found even though both displays allowed the user to freely explore the 3D data by moving through a virtual patient in physical space, as compared to the small workspace of a hand-held device like the 3D mouse. A small benefit was found for the task of tracing through real lung vessel images to a terminus. In the visualization task, spatial relations between vessel structures showed a closer relationship to true values, so that overall error was lower. Importantly, participants who were most aided in visualization by the *in situ* display were those who performed least well at the task. It seems likely that additional tasks would expand the domain in which *in situ* display is demonstrably superior.

Although the present tests constitute basic research, performed on novices without experience of visualizing medical images, our ultimate goal is to apply this method of medical image display in clinical settings, particularly in the training of novice medical personnel. Further trials should involve medical personnel at various stages of training. It would also be useful to further assess performance in clinically relevant tasks such as visual search for pulmonary nodules or classification leading to ROC curves, in addition to the spatial understanding assessed here. One venue to utilize FRISM’s intuitive display could be the anatomical laboratory, in which medical students first learn the relationships between structures in the body and their corresponding appearance on medical images. FRISM may also have clinical utility in an operating room setting, with real-time surgical path planning immediately prior to (or during) a surgical procedure. Displays on booms are already commonplace in surgical suites, although smaller, hand-held tablet computers offer an appealing alternative, eliminating the variable stiffness in the boom experienced with different poses and directions of motion. The present analysis contributes to the development of the new method of display by indicating the advantages of co-registration of data and image for the process of mentally integrating 2D data into three dimensions.



**Additional file 1:** Video of FRISM under test. (MP4 7450 kb)


## Abbreviations

2D: Two-dimensional
3D: Three-dimensional
CT: Computed tomography
FRISM: Freely moving *in-situ* medical image
IRED: Infrared emitting diode
LA: Left atrium
MR: Magnetic resonance
PA: Pulmonary artery
PET: Positron emission tomography
